# Toxic or non-toxic: a critical assessment of the contradictory results for selected graphene family materials

**DOI:** 10.3389/ftox.2026.1818582

**Published:** 2026-04-29

**Authors:** Charlene Andraos, Victor Wepener, Mary Gulumian

**Affiliations:** 1 Water Research Group, Unit for Environmental Sciences and Management, North-West University, Potchefstroom, South Africa; 2 Toxicology and Biochemistry Department, National Health Laboratory Services (NHLS), National Institute for Occupational Health (NIOH), Johannesburg, South Africa

**Keywords:** assay interference, graphene family materials (GFMs), method standardization, physicochemical characterization, toxicity

## Abstract

The toxicity of graphene, graphene oxide, and reduced graphene, selected members of graphene family materials (GFMs), has been widely investigated, yet reported biological effects remain highly inconsistent, limiting reliable hazard assessment. Here, we synthesize current evidence to examine how intrinsic material properties and methodological factors contribute to these discrepancies. Published studies were critically evaluated with particular attention to physicochemical characteristics, synthesis and post-processing histories, sonication protocols, and interactions with commonly used *in vitro* toxicity assays. The literature reveals that reported cytotoxic, genotoxic, and antimicrobial effects vary widely, ranging from pronounced dose-dependent toxicity to negligible or even beneficial biological responses. A major driver of these contradictions is pervasive interference of these selected GFMs with colorimetric, fluorometric, and luminometric assays, arising from adsorption, fluorescence quenching, optical masking, and catalytic effects, often compounded by insufficient experimental controls. Variations in oxidation state, defect density, surface chemistry, lateral size, and sonication-induced structural modification strongly influence both biological behaviour and assay artefacts, while additional confounders such as endotoxin contamination and residual impurities are rarely assessed. Overall, the evidence indicates that many contradictory toxicity outcomes reflect differences in material properties and assay interference rather than true biological inconsistency, underscoring the need for rigorous material characterisation and the adoption of interference-free or label-free testing strategies to generate reliable and reproducible toxicity data for these graphene materials.

## 1 Introduction

The toxicity of graphene (G), graphene oxide (GO), and reduced graphene (rGO), selected members of graphene family materials (GFMs) depend on different properties such as lateral dimensions, thickness, shape, surface area, layer number, oxidative state, purity, surface chemistry, surface functionalisation, and hydrophilicity or hydrophobicity resulting from synthesis-dependent oxygen content (Sanchez et al., 2012; Bussy et al., 2013; Wick et al., 2014; Ou et al., 2016). Considerable effort has therefore been devoted to determining which of these properties influence toxicity, and the effects of G, GO, and rGO on human cell lines have been widely investigated.

Current literature reports conflicting results: many studies describe unmodified G, GO, and rGO as cytotoxic or genotoxic, while others report negligible or no toxicity. For example, GO has been reported to induce toxic responses in several human cell types, including breast cancer (Wu et al., 2015), neuronal (Xiaoli et al., 2021), epithelial and macrophage (Horváth et al., 2013) cell lines. Conversely, other studies have found no obvious cytotoxicity in lung epithelial (Chang et al., 2011), fibroblast (Wang et al., 2013), or intestinal cell lines (Nguyen et al., 2015), and GO has even been reported to promote stem cell pluripotency (Chen G.-Y. et al., 2012) or enhance bacterial growth (Ruiz et al., 2011). Reviews have consequently highlighted the inconsistency of conclusions across studies (Seabra et al., 2014; Liao et al., 2018). Contradictory results are also reported for antimicrobial activity. Some studies demonstrate that GO lacks intrinsic antibacterial effects (Ruiz et al., 2011) and may even enhance bacterial growth, whereas others report strong antibacterial activity for different graphene-based materials (Liu S. et al., 2011), including GO and rGO, with suppression of bacterial viability (Hu et al., 2013) and induction of reactive oxygen species (Gurunathan et al., 2012).

Most *in vitro* toxicity studies employ dyes to assess cell viability, metabolic activity, mitochondrial integrity, and reactive oxygen species production. This review critically examines the dyes and methodologies used and evaluates how graphene materials may interfere with these test systems. To achieve this, mechanisms of commonly used assays are considered, including the intracellular or extracellular locations of dyes and their products, and whether graphene surfaces can access and interact with them. The influence of material properties such as size, lateral dimensions, layer number, C/O ratio, and hydrophilicity or hydrophobicity on assay interference is also examined, together with the effectiveness of controls used to eliminate such effects. Thus the aims of this review are to evaluate how intrinsic properties of D, GO, and rGO influence interactions and therefore the suitability of commonly used assays for toxicity assessment of these graphene materials, and outlines approaches that may reduce these inconsistencies in toxicity evaluation in future studies.

## 2 Fundamental intrinsic characteristics of G, GO, and rGO

The interaction of G, GO, and rGO with biological and environmental systems is governed by their surface properties, which in turn arise from their intrinsic characteristics. Key intrinsic characteristics of G, GO, and rGO include the presence or absence of functional groups, layer number, edges, edge and structural defects, porosity, out-of-plane disorders such as ripples, wrinkles, crumples and folds, and chirality.

### 2.1 Functional groups

**FIGURE 1:**
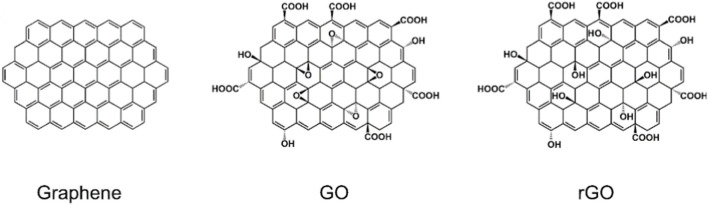
Structures of graphene, graphene oxide, and reduced graphene oxide (Geim and Novoselov, 2007). Reproduced from Zhang B. et al. (2016) under the CC BY-NC-ND license.

Intrinsic characteristics largely determine surface behaviour. Structural differences among G, GO, and rGO are illustrated in Figure 1 and have been extensively documented in the literature. Pristine G consists of a single layer of sp^2^-hybridized carbon atoms arranged in a honeycomb lattice. It is hydrophobic and therefore poorly dispersible in water, typically requiring surfactants or stabilizing agents to maintain suspension in biological media and prevent agglomeration (Taherian et al., 2013). Graphene oxide is a highly oxidized derivative of G composed of single-atom-thick sheets containing oxygen-bearing functional groups such carboxyl, epoxide, and hydroxyl groups. Edge carboxylate groups confer colloidal stability and pH-dependent negative surface charge (Park et al., 2009), while basal plane epoxide and hydroxyl groups enable hydrogen bonding and surface interactions (Kim H. et al., 2010). These functionalities allow GO to disperse readily in water and some organic solvents. Reduced graphene oxide is produced from GO by thermal, chemical, or photochemical reduction processes (Gurunathan et al., 2013; Muthoosamy et al., 2015; Zhang and Gurunathan, 2016; Szczepaniak et al., 2018). Reduction decreases oxygen content, resulting in altered surface chemistry and the formation of vacancy defects (Bagri et al., 2010). Consequently, rGO displays intermediate hydrophilicity and reactivity relative to pristine G and GO. These properties of G, GO and rGO may also determine their colloidal stability a critical aspect, which can differ according to the chosen biological assay for the toxicity assessment ((Castro et al., 2018; Franqui et al., 2019). The Derjaguin-Landau-Verwey- Overbeek (DLVO) theory may quantitatively predict the colloidal stability of GO and rGO (Gudarzi, 2016).

### 2.2 Edges

**FIGURE 2:**
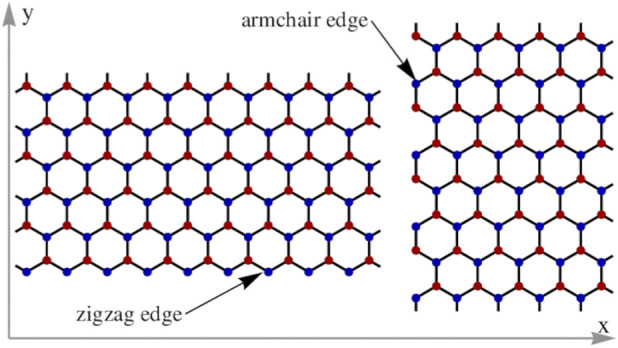
Graphene lattice with zigzag and armchair edges. Reprinted with permission from Gusynin et al., 2008. Copyright (2008) by the American Physical Society.

Dangling bonds and valence imbalances at G defects alter charge distribution and create localized unpaired electrons (Zhang L. et al., 2015), contributing to the high chemical reactivity of edges and defect sites (Wakabayashi et al., 2023). Edge reactivity is significantly greater than that of basal planes (Sharma et al., 2010). Zigzag edges contain unpaired electrons and are generally more reactive than armchair edges (Figure 2), whose bonding configuration confers greater stability (Xu and Ye, 2010). Hydrogen-terminated edges are particularly reactive, allowing anchoring of functional groups such as hydroxyl, carboxyl, hydrogen, and amine groups edges (Liu et al., 2010; Tao et al., 2016). Functionalization strategies therefore often aim to increase edge density and defect concentration, especially at zigzag sites (Loh et al., 2010a), to enhance reactivity (Brey and Fertig, 2006; Acik and Chabal, 2011; Tang et al., 2013).

Graphene oxide differs in that polar basal planes and negatively charged edge carboxylate groups enable hydrogen bonding and metal-ion complex formation (Xue et al., 2012). In rGO, oxygen removal introduces vacancy defects, reducing hydrophilicity relative to GO and lowering basal plane reactivity (Bagri et al., 2010). Edge termination, reconstruction, and functionalization also modify optical and electronic properties (Acik and Chabal, 2011). Intrinsic edges formed during exfoliation contain unsaturated carbon atoms and serve as primary sites for biochemical interactions. Oxygen-containing edge groups further alter material behaviour, making GO amphiphilic with hydrophilic edges and relatively hydrophobic graphitic basal domains (Girit et al., 2009). Oxygen incorporation sufficient to convert a substantial fraction of carbon bonds to sp^3^ hybridization further modifies edge and surface properties (Mkhoyan et al., 2009).

### 2.3 Defects and reactivity of graphene family materials

Although pristine graphene is chemically inert, materials produced through physical or chemical synthesis commonly contain defects, including Stone–Wales defects, vacancy defects, grain boundaries, holes, line defects, topological defects, and adsorbed impurities. Many originate from non-hexagonal ring formations within the lattice. During chemical vapor deposition and other synthesis processes, mismatches between growing domains generate defects, holes, and edges that increase chemical reactivity (Huang et al., 2011). Edge atoms possess localized unpaired electrons, making them particularly reactive compared with basal plane atoms (Boukhvalov and Katsnelson, 2008). Graphene’s planar structure also permits transformations from sp^2^ to sp^3^ configurations during reactions, generating additional vacancies, cracks, and impurity sites that further modify reactivity (Loh et al., 2010a).

Defects located at graphene edges or etched holes act as active adsorption and reaction sites (Acik and Chabal, 2011). Chemically reduced rGOs often contain high densities of such edge-like sites, enhancing electron transfer and biosensing performance toward small biomolecules such as NADH (Shao et al., 2010). Sheets containing Stone–Wales defects are more reactive than pristine graphene, and theoretical studies show that hydrogen-terminated zigzag edges preferentially bind certain chemical groups, e.g., thiol (– SH) (Denis, 2009), highlighting the importance of edge chemistry in adsorption and reactivity.

Structural defects formed during production strongly influence G’s electrical, structural, thermal, optical, and magnetic properties (Yue et al., 2012). Defect-free G is chemically inert and primarily interacts through weak physical adsorption (π - π interactions), contributing to reported biocompatibility in some systems (Sahni et al., 2013; Skoda et al., 2014). However, controlled introduction of defects is often required to tailor graphene properties for specific applications (Tian et al., 2017; Yang et al., 2018). Defects may arise spontaneously, during synthesis, or through intentional modification. They alter electronic structure, chemical susceptibility, charge transport, and may introduce impurities into the carbon lattice by substitution or functionalization of some original carbon atoms (Gao et al., 2011; Araujo et al., 2012). While high-quality G relies on low defect density, large-scale production typically introduces curvature, holes, cavities, and edge irregularities. Even low defect concentrations significantly modify material behaviour, and graphene membranes naturally develop ripples and distortions that further contribute to structural disorder (Fasolino et al., 2007).

### 2.4 Porosity of graphene family materials

**FIGURE 3:**
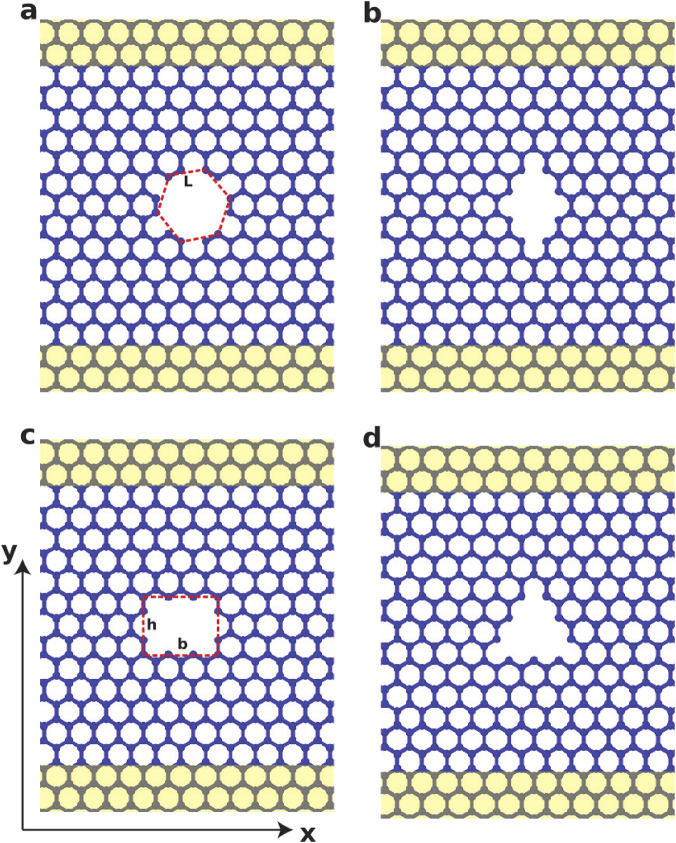
Atomic configuration of nanoporous graphene membranes with pores: **(a)** hexagonal, **(b)** rhombic, **(c)** rectangular and **(d)** triangular. The yellow regions are fixed to uniaxially strain the nanoporous graphene membranes in the armchair direction (parallel to the “y” axis). Similarly in the zigzag direction. Reproduced from Cabanillas-Casas et al. (2021) under the CC BY-ND license.

Porous graphene contains nanopores within the basal plane, with pore size and distribution determined by production method (Xue et al., 2012). Pores may exhibit various geometries (e.g., hexagonal, rhombic, rectangular, or triangular shapes), forming nanoporous graphene membranes (Cabanillas-Casas et al., 2021) (Figure 3).

Based on diameter, pores are classified as micropores (<2 nm), mesopores (2–50 nm), or macropores (>50 nm) (Zdravkov et al., 2007). Porosity alters graphene properties and underpins numerous applications (Xue et al., 2012). Pore distribution and volume determine performance in specific uses (Yang et al., 2017). In-plane pores can be introduced through focused electron (Fischbein and Drndić, 2008; Fox et al., 2011) or ion beam (Celebi et al., 2014) techniques, UV oxidative etching (Koenig et al., 2012) or ion bombardment followed by chemical etching (O’Hern et al., 2014). These pores generate internal edges that increase surface reactivity and surface area, enhancing adsorption and catalytic behaviour (Jabari Seresht et al., 2013).

Out-of-plane disorders such as ripples, wrinkles, crumples, and folds arise during growth and transfer processes (Fasolino et al., 2007; Liu N. et al., 2011; Deng and Berry, 2016; Sun et al., 2023; Wang, 2023). Localized variations in bond lengths and thermal fluctuations drive graphene away from perfect planarity, producing corrugations in graphene sheets (Xu P. et al., 2014) observable by scanning tunnelling microscopy (Deng and Berry, 2016). Ripple patterns depend on sheet size, temperature, and substrate interactions. Cooling-induced thermal mismatch with substrates generates wrinkles (Obraztsov et al., 2007), while multidirectional forces during evaporation produce crumpling (Deng and Berry, 2016). Physical and chemical environments, vacancies, sp^3^ bonding, and adsorbates can also induce surface corrugation. Although often undesirable due to their impact on properties, several approaches can reduce such disorder, including deposition of graphene on smooth substrates (Lui et al., 2009), optimized transfer procedures, or strain-controlled manipulation using polymer supports (Zang et al., 2013).

### 2.5 Chirality

Chirality arises when structures are not superimposable on their mirror images, producing enantiomeric forms with similar physicochemical properties but distinct biological interactions (Cintas, 2007). Chirality in nanomaterials may originate from adsorption of chiral molecules (extrinsic) or from intrinsic surface structures such as kinked or stepped features. Chiral surfaces influence adsorption, cellular uptake, intracellular distribution, and toxicity through stereoselective interactions with biological molecules (Utembe, 2019). Graphene can acquire chirality through functionalization with chiral molecules, including amino acids, often enhanced by sonication-assisted processes (Pranav et al., 2023). Intrinsic chirality may also arise from twisted stacking, buckling (Evans et al., 2018), and edge configurations (Herbut et al., 2009; Koshino et al., 2014). Such features can be detected using spectroscopic methods, including Raman absorption spectroscopy (Qiu et al., 2024), and are increasingly exploited in biomedical and pharmaceutical applications, although associated toxicological implications remain under investigation (Zhao et al., 2021).

## 3 Influence of intrinsic characteristics on properties of G, GO, and rGO

The foregoing intrinsic structural features impart distinct physicochemical properties to G, GO, and rGO that influence their interactions in biological and environmental systems.

### 3.1 Optical properties

Single-layer graphene transmits approximately 97.7% of incident light across a broad wavelength range (Nair et al., 2008), while absorption and optical contrast increase with increasing layer number (Kravets et al., 2010). For GO, optical absorption and surface charge can be tuned through preparation conditions (Kang and Suk, 2012). Graphene oxide exhibits strong absorption within the visible range (380–800 nm) and slightly reduced absorption in the ultraviolet region, indicating good photo-response in both visible and UV regions and supporting its potential in light-based applications (Song et al., 2014).

### 3.2 Fluorescence

Fluorescence behaviour in G, GO, and rGO depends largely on electronic structure, which changes with oxidation level and sheet size (Geim and Novoselov, 2007; Shang et al., 2012). Pristine G, composed entirely of sp^2^ carbon, exhibits negligible fluorescence due to its zero band gap (Zhu et al., 2010). In contrast, GO and rGO contain mixed sp^2^/sp^3^ carbon networks created by oxygen functional groups, opening an optical band gap and enabling fluorescence (Shang et al., 2012; Vempati and Uyar, 2014). Graphene oxide fluorescence arises from electron–hole recombination involving transitions between non-oxidized sp^2^ carbon domains and surrounding oxidized regions. Emission typically occurs over a broad wavelength range and depends on excitation wavelength, oxidation state, and environmental conditions (Shang et al., 2012). Upon excitation the protonation of COO^−^ to COOH under acidic conditions generally reduces fluorescence intensity, whereas basic conditions may restore emission through deprotonation effects moieties (Loh et al., 2010b; Galande et al., 2011; Mei et al., 2019).

Fluorescence properties are further influenced by sheet size (Kim et al., 2020) and the degree of reduction (Twardowska et al., 2014); increased reduction usually decreases fluorescence intensity while shifting emission peaks (Kumar et al., 2014). Graphitic domain abundance, reflected by C/O ratio, also affects fluorescence quenching efficiency (Swathi and Sebastian, 2008), with less oxidized GO (i.e., C/O = 1.9) typically exhibiting stronger quenching due to larger graphitic domains (Hong et al., 2012).

### 3.3 Adsorptive properties

Graphene oxide interacts strongly with dyes and other molecules through π–π stacking (Xiao et al., 2016), electrostatic interactions (Minitha et al., 2017), and hydrogen bonding (Chen D. et al., 2012). Due to abundant oxygen-containing functional groups and large surface area, GO typically exhibits higher adsorption capacity than rGO or pristine G. Graphene adsorption relies mainly on non-covalent interactions, while rGO displays intermediate adsorption performance for both cationic and anionic dyes (Mounra et al., 2023). Graphene oxide sheets carry an overall negative charge due to deprotonation of surface functional groups such as hydroxyl (−OH), carboxyl −COOH) and epoxy (−COC −) groups (Li et al., 2008). This not only facilitates adsorption but also promotes irreversible aggregation (Wang et al., 2016) when cations or cationic dyes neutralize surface charges (Szabo et al., 2020). Adsorption efficiency depends strongly on oxidation degree, sheet size, and distribution of oxidized versus graphitic domains (Tan et al., 2017; Lee et al., 2019). Highly oxidized GO often shows increased affinity for hydrophilic species, whereas graphitic regions preferentially bind hydrophobic compounds (Yan et al., 2015).

Physical adsorption processes may also result in fluorescence dye quenching. Graphene oxide can function both as a fluorophore and an efficient quencher (He et al., 2015; Zheng and Wu, 2017; Basko et al., 2020) via charge transfer or resonance energy transfer mechanisms (Smitha et al., 2020). Quenching efficiency is influenced by GO concentration, oxidation state, sheet size, fluorophore charge, pH, temperature, and distance between fluorophore and graphene surface (Hu et al., 2013; Srisantitham et al., 2018; Ferreira et al., 2024). Surface modifications or polymer functionalization can enhance fluorescence or tailor sensing properties (Gao et al., 2019; Wang et al., 2019).

Adsorption of probe dyes onto graphene materials is a known source of artifacts in toxicity assays, as optical and adsorptive interference varies with material surface chemistry, hydrophobicity, and layer number (Creighton et al., 2013). Materials with fewer layers, i.e., less thickness, smaller specific surface area, and lower bending elasticity (Wick et al., 2014; Dasari Shareena et al., 2018) and more hydrophobic surfaces tend to exhibit stronger interference. Layer number also influences adsorption through changes in thickness, stiffness, and specific surface area. Monolayer graphene and GO possess extremely high theoretical surface areas (Stoller et al., 2008), making surface interactions dominant in biological responses. As layer number increases, specific surface area decreases, reducing adsorption capacity, while thicker materials may exhibit increased rigidity during cellular interactions (Bhattacharya et al., 2016).

### 3.4 Photoluminescence

Photoluminescence in GO results from radiative recombination of electron–hole pairs within isolated sp^2^ carbon domains embedded in an sp^3^ carbon–oxygen matrix (Eda et al., 2010). Progressive reduction of GO enlarges sp^2^ domains, gradually transforming GO toward graphene-like structures and consequently reducing or eliminating photoluminescence (Ding et al., 2018).

### 3.5 Ability to form covalent and non-covalent interactions

Surface functionalisation of graphene materials is commonly achieved via (1) covalent modification, typically involving oxygenated groups, and (2) non-covalent modification through intermolecular interactions (Boukhvalov and Katsnelson, 2008) such as electrostatic forces and van der Waals interactions. Covalent functionalisation involves reactions between oxygen-containing groups on GO/rGO and functional groups of target molecules, whereas non-covalent functionalisation relies on weak interactions that preserve the carbon framework and is generally simpler to perform (Ismail et al., 2021).

Covalent interactions occur at short separation distances (2 – 3 A) and involve strong orbital overlap. Covalent functionalisation—particularly for GO and rGO—disrupts the π-conjugated system and may therefore alter key properties such as electrical conductivity and mechanical performance (Plachá and Jampilek, 2019). Surfaces of GO contains hydroxyl, epoxide, and carboxyl groups (Chua and Pumera, 2013), with edge carboxyl groups being especially reactive, enabling diverse routes for covalent modification (OuYang et al., 2008; Choi et al., 2018). Functionalisation may also occur through reactions involving aromatic C=C bonds (Sinitskii et al., 2010) or through hydroxyl-based chemistry (e.g., esterification or amidation) (Xiong et al., 2012).

Polymeric functionalisation is widely used to increase dispersibility and stability in biological media (Sun et al., 2010). Polymers bearing reactive groups (e.g., amines and hydroxyls) such as polylysine, polyethylene glycol (PEG), polyvinyl alcohol, and polyallylamine can improve dispersion stability and modify morphology (Khan et al., 2019). PEG, poly (vinyl alcohol), chitosan, and related coatings may also modulate biocompatibility (Xu Z. et al., 2014; Pinto et al., 2016). In addition to intentional modifications, graphene materials may undergo unintended functionalisation through adsorption of biomolecules (corona formation) in biological systems (Di Santo et al., 2020) or through interactions following environmental release (Nasser et al., 2020). Because covalent bonding occurs readily at edges and defects (Konatham and Striolo, 2009), functionalisation strategies have also been used to reduce reported toxicity by conjugating graphene materials with biocompatible polymers such as PEG, PEI, or chitosan (Sasidharan et al., 2012; Chatterjee et al., 2014).

Non-covalent interactions involve physisorption and larger separation distances (3–5 A) than covalent bonds, resulting in weaker and less stable modifications under *in vitro* and *in vivo* conditions (Tiwari et al., 2020). Nevertheless, the high G surface area and delocalized π-electron system support extensive non-covalent adsorption-based modification (Qamar et al., 2024). Overall, surface functionalisation is commonly employed to improve dispersion, reduce agglomeration, and preserve desirable material properties (Wang et al., 2013), and its role in modulating graphene toxicity has been reviewed (Guo et al., 2021).

### 3.6 Ability to produce free radicals

The type and abundance of oxygenated functional groups (e.g., carboxyl, hydroxyl, epoxide, carbonyl) depend on GO preparation and oxidation extent and influence defect density, sheet size, and electronic structure (Gao et al., 2009; Dreyer et al., 2010). These features contribute to formation of isolated sp^2^ domains and altered electron distribution, enabling localisation of unpaired electrons (radicals). Extensive oxidation may also generate holes (internal edges), whose edge geometry and chemistry can create radical-like edge states (Fujii et al., 2014). Reduction of GO generally increases defects and local electrochemical activity; however, evidence suggests that mildly reduced GO may exhibit higher radical content and reactivity than more extensively reduced materials (Komeily-Nia et al., 2020; 2021). In biological systems, GO has been reported to induce greater reactive oxygen species (ROS) production than few-layer graphene (Pelin et al., 2018), consistent with the higher density of oxygen-containing groups that can promote ROS generation (Zou et al., 2016). Oxidized graphene derivatives have accordingly been associated with higher ROS levels than pristine graphene (Majeed et al., 2017), highlighting the central role of surface chemistry in oxidative responses (Georgakilas et al., 2012; Chua and Pumera, 2013).

### 3.7 Catalysis

Graphene, GO, and rGO can exhibit catalytic activity toward adsorbed dyes via adsorption-assisted catalysis, photocatalysis, and charge transfer processes, supporting applications in wastewater treatment and environmental remediation (Singh et al., 2020; Kanwal et al., 2023). rGO-based composites have been used to degrade dye molecules (Ucar et al., 2017), and relationships between rGO morphology, specific surface area, oxygen-containing groups, and catalytic/adsorptive performance have been reported (Bai et al., 2013; Liu S. et al., 2014). Structural features such as nanoripples may also contribute to catalytic activity (Wang, 2023).

In photocatalysis, irradiation generates electron–hole pairs that drive formation of reactive oxygen species (e.g., superoxide and hydroxyl radicals), which degrade dye molecules (Singh et al., 2020). Photocatalytic performance is strongly influenced by surface area (Sandoval et al., 2017), where alkaline conditions can enhance hydroxyl radical generation (Yang et al., 2011) and increase degradation rates. rGO interacts with dyes through π–π interactions, electrostatic forces, structural conjugation, and hydrophobic associations, supporting adsorption and subsequent catalytic transformation (Mahich et al., 2024).

## 4 Summary of relationship between intrinsic factors and G, GO, and rGO properties

For meaningful assessment of G, GO, and rGO toxicity, reporting should extend beyond general physicochemical descriptors to include intrinsic properties such as oxidation level; functional group distribution and clustering; edge type; defect and nanopore density; out-of-plane disorder (ripples, wrinkles, crumples, folds); and chirality. These intrinsic features govern material behaviour in biological and environmental matrices and, critically, determine the likelihood of surface interactions with assay substrates and products. Consequently, assay interference is a major concern when applying *in vitro* methods originally developed for soluble chemicals. The importance of interference across assay systems is therefore examined in the following sections.

## 5 Application of dyes in graphene family material toxicity assessment and the interaction with surfaces of materials based on their properties

*In vitro* cell cultures are widely used to assess the toxicity of G, GO, and rGO, employing numerous colorimetric, fluorometric, luminometric, and dye-exclusion assays. These assays can be classified by measured endpoints reflecting different cellular functions and detection mechanisms. Given the strong surface activity of G, GO, and rGO, it is essential to critically evaluate dye-based assays used in the literature and consider whether experimental conditions promote interactions between these graphene material surfaces and assay substrates or products, thereby generating interference and altering reported toxicity outcomes. Accordingly, the following sections summarise key experimental variables of the most commonly used assays relevant to interference, including cell type, synthesis method, and dispersion procedures (including sonication), as well as the extent and quality of material characterisation reported by investigators.

## 6 Dye exclusion assays

### 6.1 Trypan blue membrane integrity assay

Trypan blue is a negatively charged exclusion dye used to assess cell membrane integrity. Viable cells exclude the dye and retain a clear cytoplasm, whereas non-viable cells permit dye entry and appear blue. Typically, cells are exposed to test materials, washed, suspended in trypan blue solution, and viable and non-viable cells are counted microscopically (Strober, 2015). However, trypan blue cannot distinguish between fully functional cells and those that remain viable but are functionally compromised, limiting its sensitivity for cytotoxicity testing. In addition, trypan blue itself may exert toxic effects on mammalian cells (Kim et al., 2016).

Studies (see Table 1 for relevant studies and references) employing the trypan blue assay have reported conflicting toxicity results for G, GO, and rGO. Some investigations observed no mortality in exposed cells, irrespective of GO size or concentration (Chang et al., 2011; Malkova et al., 2021), while others reported dose-dependent reductions in viability across several cell lines (Liao et al., 2011; Zhang and Gurunathan, 2016). In certain studies, larger GO sheets produced stronger membrane damage than smaller sheets (Vranic et al., 2018), whereas modified or reduced materials sometimes produced weaker or no cytotoxic responses (Gurunathan et al., 2013; Zhang and Gurunathan, 2016). These results indicate substantial variability across material types, sizes, and experimental systems. Reported synthesis routes also vary widely, with many studies using GO produced via modified Hummers’ methods, followed by size separation through centrifugation or sonication, while others employed commercially obtained materials with limited synthesis information. Dispersion methods also differed considerably, with some studies applying prolonged sonication, others using vortexing, and several providing limited procedural detail. Such variations likely influence particle size distributions and dispersion stability, contributing to inconsistent outcomes.

**TABLE 1:** Dye exclusion assays used to determine *in vitro* toxicity of different graphene family materials.

Assay	Material	Synthesis method	Comments	Cell line tested	Toxicity	References
Trypan Blue	G and GO	Commercial - not specified	Dispersion (sonication in ultra-pure water for 30 min)	HDF	Yes – dose dependent	Liao et al. (2011)
	G	Commercial - not specified	Dispersion - none	U87-MG, U118-MG	Yes – cell line difference	Jaworski et al. (2013)
	GO	modified Hummer’s – further heating and centrifuged for different sizes	Dispersion (sonication – 1 h)	A549	None	Chang et al. (2011)
	GO	Commercial - not specified	Dispersion (sonication in 0.02% sodium cholate for 30 min)	TPH-1	None	Malkova et al. (2021)
	GO	modified Hummer’s – sonicated and centrifuged for different sizes	Dispersion - vortex	BEAS-2B	Yes – size effect	Vranic et al. (2018)
	GO, B-rGO	modified Hummer’s – bath and probe sonication	Reduction – biological (*B. marisflavi*)	MCF-7	Yes	Gurunathan et al. (2013)
	G, GO, rGO	Commercial - not specified	Dispersion (sonication in ultra-pure water for 30 min)	U87 - MG	Yes	Hinzmann et al. (2014)
	GO, rGO	Hummer’s	Reduction (nicotinamide)	MEF	Yes – dose dependent	Zhang and Gurunathan (2016)
Propidium iodide/Annexin V	GO, rGO	Hummer’s	Reduction - chemical Dispersion – G none; GO and rGO - sonication 45 min	U87-MG, U118-MG	Induced apoptosis and necrosis in U87	Jaworski et al. (2015)
	GO	modified Hummer’s – sonicated and centrifuged for different sizes	No information	BEAS-2B	Induced apoptosis but limited necrosis	Vranic et al. (2018)
	G, GO	Arc discharge for G and nitric acid for GO	No information	RAW 264.7	G induced apoptosis	Sasidharan et al. (2012)
	GO, rGO	Hummer’s	Reduction – chemical with hydrazine Dispersion – aqueous no time indicated	RAW 264.7, A549	Limited induced by GO	Horváth et al. (2013)
	GO, GO- colloids	Commercial – not specified	No information	A549	None	Domi et al. (2019)
	Few layer G, GO	Ball-milling for G and commercial GO – not specified	No information	HaCaT	Only in GO	Pelin et al. (2017)
	G, rGO	G obtained commercially	Reduction – chemical and heat Dispersion (sonication in ultra-pure water for 30 min)	U87-MG, U118-MG	Only in G	Szczepaniak et al. (2018)

Graphene materials may remain associated with cell membranes even after washing, allowing residual particles to interact with trypan blue and interfere with viability assessment (Jaworski et al., 2013; Vranic et al., 2018). Graphene particles themselves may stain or scatter light similarly to dye-stained dead cells, potentially leading to overestimation of cell mortality (Altman et al., 1993). In addition, serum proteins can bind trypan blue, producing misleading staining patterns. Extended dye exposure may also cause gradual uptake by viable cells, further complicating interpretation (Strober, 2015).

### 6.2 Annexin V/propidium iodide (PI) apoptosis assay and calcein-acetoxymethyl (AM)/PI assay

Propidium iodide (PI) is a nucleic acid-binding exclusion dye that stains cells with compromised membranes, allowing discrimination between viable and dead cells (Johnson et al., 2013). Annexin V assays detect apoptosis by binding phosphatidylserine (PS), which becomes externalized early in apoptosis. Annexin V is commonly conjugated to fluorescent dyes, enabling detection of early apoptotic cells (Annexin V positive, PI negative), while combined Annexin V and PI staining identifies late apoptotic or necrotic cells. Viable cells show minimal Annexin V staining (Horváth et al., 2013). Studies using Annexin V/PI assays have shown variable responses across cell types and graphene materials. Some investigations report apoptosis as the predominant mechanism of cell death, often dependent on material size, oxidation state, or surface chemistry (Jaworski et al., 2015). Comparative studies frequently show differing toxicity between pristine graphene, GO, and rGO, with surface functionalization sometimes reducing apoptotic responses, while highly oxidized materials or specific rGO forms induce stronger effects (see Table 1). Differences in synthesis routes, reduction methods, and material sourcing are widely reported across studies. Sample preparation procedures also vary, with inconsistent reporting of sonication or dispersion conditions (Table 1). Such methodological variability likely contributes to differences in observed cellular responses.

Physical adsorption of PI or fluorophore-labelled Annexin V onto graphene surfaces may result in fluorescence quenching (Treossi et al., 2009; Kim J. et al., 2010; Liu F. et al., 2014). Graphitic domains, particularly abundant in less oxidized materials (C/O ratio = 1.9), are known to quench fluorescence via energy transfer mechanisms (Swathi and Sebastian, 2008). Consequently, reduced fluorescence signals may not solely reflect biological responses but may also arise from dye quenching by graphene materials (Kasry et al., 2012; Pustovit and Shahbazyan, 2012; Domi et al., 2019). Variations in oxidation state and C/O ratio influence quenching efficiency, further complicating comparisons between materials (Kasry et al., 2012). Furthermore, as demonstrated by Bohmer et al. (2018) using the Annexin V/PI assay and by Malina et al. (2021) using the calcein-AM/PI Live/Dead assay with flow cytometry is subject to substantial particle interference. These interferences manifest as intrinsic fluorescence, fluorescence quenching, and the formation of free nano-agglomerates that shift cellular scatter profiles and mimic dead cell populations. While it is possible to mitigate these effects through the implementation of rigorous “spike-in” controls, where particles are added to control cells immediately prior to measurement, and the establishment of specific particle-positive gating strategies, this process is tedious and technically demanding. The requirement for manual, multi-step compensation and the use of specialized orthogonal controls for each specific material makes these methods difficult to standardize for predictive toxicology. Consequently, even though these sophisticated protocols can provide a more reliable dataset, they rely on labour-intensive correction rather than the elimination of interference.

## 7 Colorimetric assays

Colorimetric assays assess cell viability indirectly by measuring biochemical markers of metabolic activity. Viability reagents generate a colour change proportional to the number of metabolically active cells, which is quantified spectrophotometrically (Präbst et al., 2017).

### 7.1 Tetrazolium reduction assays

Tetrazolium reduction assays are among the most widely used viability tests for G, GO, and rGO. Common tetrazolium salts include MTT, MTS, XTT, WST-1, and WST-8. These assays rely on the reducing environment of metabolically active cells, where tetrazolium salts are converted to coloured formazans, largely linked to cellular dehydrogenase activity and reducing equivalents, e.g., NADH-associated processes (Berridge and Tan, 1993; Chamchoy et al., 2019). A key distinction is that MTT produces an insoluble formazan requiring a solubilisation step, whereas MTS, XTT, WST-1, and WST-8 generate water-soluble formazans. Given their frequent use in graphene toxicology, MTT, WST-1, and WST-8 are highlighted below.

### 7.2 MTT assay

The MTT assay measures the conversion of yellow MTT to insoluble purple formazan by viable cells. After exposure to test materials, MTT is added, reduced intracellularly, and the resulting formazan crystals are solubilised (commonly with DMSO) for absorbance measurement (typically ∼570 nm with 655 nm as reference wavelength).

MTT has been widely applied to graphene materials, but published outcomes are inconsistent across materials, cell types, and experimental conditions (see Table 2). Several studies report dose- and time-dependent cytotoxicity for G, GO, or rGO (Srikanth et al., 2018), whereas others report little or no toxicity in comparable systems (e.g., Horváth et al., 2013). Functionalisation (e.g., PEG- or PEI-based coatings) is frequently associated with reduced toxicity, consistent with altered surface chemistry and charge (Wang et al., 2013; Syama et al., 2016). Across studies, variability in material sourcing/synthesis, oxygen functional group content, and dispersion protocols (including sonication) is substantial and may contribute to divergent results and comparability limitations.

**TABLE 2:** Tetrazolium reduction assays used to determine *in vitro* toxicity of different graphene family materials.

Assay	Material	Synthesis method	Comments	Cell line tested	Toxicity	References
MTT	G	Not specified	​	PC12	Yes	Zhang et al. (2010a)
	GO	Sonication at 40 kHz for 1 h	​	BF-2	Yes – dose dependent	Srikanth et al. (2018)
	GO, PEG-rGO	Not specified	PEG functionalized GO	HLF	Yes – reduced with PEG groups	Wang et al. (2013)
	GO, PEG-rGO	Sonication - not specified	Reduction - hydrazine	MSC	None for GO and increased viability with rGO	(Syama et al., 2016)
	GO, rGO	Sonication – rigorous at 40 kHz	​	HUVEC, HaCaT, MG63	Yes – rGO less toxic	Das et al. (2013)
	G, rGO	Sonication- gently for 30 min in ultra-pure water	​	Hs-5, U87-GM	None for G and rGO with largest surface area most toxic	Szczepaniak et al. (2018)
	GO, rGO	Sonication - not specified	​	A549, RAW246.7	None	Horváth et al. (2013)
	GO	Sonication for 3 h at 42 kHz	​	CACO-2	None	Nguyen et al. (2015)
	GO	Not specified	​	A549	​	Domi et al. (2019)
WST-1	GO, rGO	Hummer’s	Functionalization with PEG, Pluronic P123, DOC	L929	P123 most toxic with PEG-rGO least toxic	Wojtoniszak et al. (2012)
	G, GO	Not specified	GO with functionalized O_2_-groups	PC12	Toxicity decreased with increasing O_2_-groups	Majeed et al. (2017)
	G	Not specified	Dispersion - sonic probe for 30 min with a 65% amplitude	PAEC from C57BL	Dose and time dependent toxicity	Bavorova et al. (2022)
WST-8	GO	Hummer’s	​	HEK293	Yes	Gurunathan et al. (2019)
	GO	Commercial – not specified	Dispersion – 30 min at 50 W	SH-SY5Y	Yes	Xiaoli et al. (2021)
	GO	Hummer’s, heated to obtain different sizes	Dispersion: 1 h at 40 kHz	A549	Yes – size dependent	Chang et al. (2011)
	Few layer G, GO	Commercial – not specified	Reduction – chemical and heat Dispersion (sonication in ultra-pure water for 30 min)	HaCaT	GO (concentration dependent) more toxic than G	Pelin et al. (2017)
	G, GO	Hummer’s, bath or probe sonication at different time intervals for different sizes	Dispersion – bath and probe sonication	NHDF	Yes	Liao et al. (2011)
	GO, rGO	Hummer’s	Reduction – biological with *B. marisflavi*	MCF-7	Yes	Gurunathan et al. (2013)
	GO, rGO	Hummer’s	Reduction - nicotinamide	MEF	Yes for GO but none for rGO	Zhang and Gurunathan (2016)
	GO	Commercial – not specified	Dispersion – 3 h at 42 kHz	CACO-2	Yes but limited	Nguyen et al. (2015)
	rGO	Hummer’s	Dispersion – 45 min with no other information	NHDF	Yes	Chortarea et al. (2022)
	GO	Hummer’s	Reduction – thermochemically Dispersion – 45 min at 44 kHz	16HBE14o	Yes with lower C/O ratios	Rodríguez-Garraus et al. (2023)

### 7.3 WST-1 assay

Reports differ regarding whether WST-1 reduction occurs extracellularly or intracellularly. One view is that WST-1 remains extracellular due to its net negative charge and is reduced via plasma-membrane-associated electron transfer involving intermediates (Berridge et al., 2005), while another describes mitochondrial reduction in metabolically active cells, generating a soluble formazan proportional to viable cell number (Wojtoniszak et al., 2012). WST-1 and its formazan are generally described as stable and of low cytotoxicity.

A limited number of graphene studies have employed WST-1. Available reports indicate variable cytotoxicity patterns across functionalised and non-functionalised GO/rGO and across materials spanning low-to-high oxygen content, supporting the importance of surface chemistry (see Table 2). Differences in synthesis provenance and dispersion methods (including inconsistent sonication reporting) further limit direct comparisons.

### 7.4 WST-8 assay

The WST-8 operates on the same general principle as other tetrazolium assays but uses a highly water-soluble, disulfonated tetrazolium salt that yields an orange, soluble formazan. After exposure, WST-8 is added and absorbance is measured at commonly ∼450 nm with 655 nm with a reference wavelength (Tominaga et al., 1999).

WST-8 is widely used in graphene toxicology literature and, as with MTT, yields conflicting results. Numerous studies report cytotoxicity of GO and/or rGO across different cell lines, with effects sometimes dependent on concentration, size fraction, oxidation state, or C/O ratio (see Table 2). Other studies report only mild or negligible toxicity under comparable exposure conditions (Nguyen et al., 2015; Zhang and Gurunathan, 2016). Differences in GO synthesis (commonly Hummers-based methods, though not always fully described), reduction strategies for rGO, and dispersion protocols (especially sonication intensity/duration) are frequently noted and likely contribute to variability.

### 7.5 Interference with tetrazolium reduction assays

Graphene materials can interfere with tetrazolium assays through adsorption of assay substrates and/or formazan products, optical artefacts, electron transfer effects, and in some reports catalytic conversion of tetrazolium salts. Adsorption mechanisms include π–π and electrostatic interactions, and adsorption strength may differ between dyes (e.g., depending on the extent of π-conjugation) (Liao et al., 2011). Cell-free reduction and/or adsorption of formazan onto graphene surfaces may produce misleading signals, potentially yielding false estimates of viability.

Adsorption behaviour has been demonstrated for both hydrophilic GO (high oxygen content) and hydrophobic rGO (low oxygen content), with material-specific interactions affecting uptake of both substrate and product (Jiao et al., 2015; Domi et al., 2019; Ćetković, T. et al., 2022). Some studies report no catalytic conversion under their conditions, illustrating that interference mechanisms may depend on graphene type, surface chemistry, and assay workflow (Horváth et al., 2013). Overall, these findings highlight that tetrazolium assays may produce unreliable results for graphene materials unless interference is rigorously controlled.

## 8 Fluorometric assays

Fluorometric assays were developed as alternatives to dye exclusion and colorimetric methods. They typically rely on intracellular conversion of a non-fluorescent precursor (e.g., esterase-cleaved probes) into a fluorescent product, with the signal used as a proxy for viable cell number or metabolic status. These assays are generally more sensitive than colorimetric tests and are applicable to adherent and suspension cultures (Page et al., 1993; O’Brien et al., 2000), but fluorescence interference from test materials is a known limitation (Altman et al., 1993; Riss et al., 2004).

### 8.1 Lactate dehydrogenase (LDH) membrane integrity assay

The LDH assay measures loss of membrane integrity via release of cytosolic LDH into culture media. LDH catalyses lactate-to-pyruvate conversion with concomitant NADH oxidation (Korzeniewski and Callewaert, 1983; Sasaki et al., 1992), and assay systems commonly couple this to tetrazolium reduction (e.g., INT) or resazurin to produce a coloured formazan or a fluorescent product (e.g., resorufin) proportional to LDH activity. Because LDH content varies by cell line, optimisation of cell number is recommended (Kumar et al., 2018). According to Holder et al. (2012) nanoparticle-mediated inhibition of LDH activity and other assay limitations have been reported.

LDH-based studies of graphene materials report mixed outcomes (see Table 3), with some observing LDH release only at higher concentrations (Zhang Y. et al., 2010) and others reporting dose-dependent membrane damage for GO and/or rGO (Gurunathan et al., 2013; 2019). Conversely, several studies report minimal or no LDH leakage under their tested conditions (Kucki et al., 2018; Malkova et al., 2021). As with other assays, reported synthesis methods, material provenance, and dispersion procedures vary substantially, and sonication is inconsistently described.

**TABLE 3:** Colorimetric assays used to determine *in vitro* toxicity of different graphene family materials.

Assay	Material	Synthesis method	Comments	Cell line tested	Toxicity	References
Lactate dehydrogenase (LDH)	G	Radio frequency catalytic chemical vapor deposition (RF-cCVD)	​	PC12	Yes at high concentrations	Zhang et al. (2010a)
	G	Arc discharge	​	RAW 264.7	Yes – dose dependent	Sasidharan et al. (2012)
	G	Commercial - not specified	Dispersion - sonic probe with 65% amplitude	PAEC	Yes	Bavorova et al. (2022)
	rGO	Sonication - not specified	Reduction – hydrazine hydrate	HUVEC	Yes with increasing C/O ratio	Das et al. (2013)
	rGO	Hummer’s	Dispersion - sonication for 45 min	THP-1, RAW246.7	Yes with increasing C/O ratio	Chortarea et al. (2022)
	G	Commercial - not specified	Dispersion – sonication for 30 min	U87-GM, U118-GM	Greater toxicity in U87 cells	Jaworski et al. (2013)
	GO	Hummer’s	​	HEK293	Yes – dose dependent	Gurunathan et al. (2019)
	GO, B-rGO	Hummer’s	Reduction - nicotinamide	MCF-7	Yes – dose dependent	Gurunathan et al. (2013)
	GO	Hummer’s	​	MEF	Yes	Zhang and Gurunathan (2016)
	GO	Hummer’s	​	BEAS-2B	Yes – size dependent	Vranic et al. (2018)
	GO	Hummer’s	Dispersion - sonication for 1 h (40 kHz, 50 W)	A549	No	Chang et al. (2011)
	GO	Hummer’s	Dispersion – bath sonication for 1 min	Caco-2 cells	No	Kucki et al. (2016)
	GO	Hummer’s	​	BeWo	No	Kucki et al. (2018)
	G	Commercial - not specified	Dispersion –sonic probe with 65% amplitude for 30 min	THP-1	No	Malkova et al. (2021)
Neutral red uptake (NRU)	GO	Commercial - not specified	Dispersion - sonication for 1 h (40 kHz, 50 W)	BF-2	Yes – dose dependent	Srikanth et al. (2018)
	GO	Hummer’s - derived from helical-ribbon carbon nanofibers (GANF 70% graphitization degree) by chemical oxidation	Dispersion - sonic probe for 20 min	PLHC-1, CLC	Yes – more toxic and time dependent in PLCHC-1	Kalman et al. (2019)
	GO	Hummer’s	Dispersion - bath sonication at 37 kHz for 2 h	CXYG	Yes – dose dependent	Lammel et al. (2013)
	GO	Commercial – not specified	​	A549	No	Domi et al. (2019)
	GO, rGO	Commercial - not specified	​	HT-29	No	Muthoosamy et al. (2015)

Interference has been raised as a concern for LDH assays with graphene materials (Majeed et al., 2017). Proposed strategies to mitigate interference include measuring LDH in cell lysates after pelleting graphene materials (Ali-Boucetta et al., 2011). However, because LDH detection relies on enzymatic reactions producing coloured formazan, graphene interactions with substrates and/or reaction products may still occur and compromise readouts. Adsorption of LDH by graphene materials has been reported and would be expected to underestimate LDH activity (Liao et al., 2011). In addition, approaches that omit key reaction components (e.g., NADH-dependent conversion steps) cannot reliably evaluate interference with formazan generation. Overall, the available evidence supports that graphene–assay interactions may yield false-negative or otherwise biased LDH results, particularly when internalisation profiles differ among graphene materials.

### 8.2 Neutral red uptake (NRU) membrane integrity assay

The NRU assay estimates viable cell number based on the ability of healthy cells to uptake and retain neutral red in lysosomes, reflecting maintenance of pH gradients and ATP-dependent lysosomal function (Repetto et al., 2008). Neutral red crosses membranes in its uncharged form and becomes protonated and trapped in lysosomes; loss of viability or disrupted pH gradients reduces retention. Dye is subsequently extracted and quantified spectrophotometrically or fluorometrically.

NRU studies report variable toxicity patterns for GO and rGO (see Table 3), including dose-dependent declines in viability in some systems (Srikanth et al., 2018), cell-line-specific sensitivity at equivalent concentrations (Kalman et al., 2019), and negligible toxicity in others (Domi et al., 2019). Differences in GO sourcing/synthesis (often Hummers-derived or commercially sourced) and dispersion methods are again inconsistently reported.

Fluorescence-based NRU measurements may be affected by graphene-mediated quenching through sorption of the fluorophore to graphene surfaces (Lammel et al., 2013). Dose-dependent attenuation of fluorescence has been reported for GO and carboxylated graphene, consistent with quenching driven by adsorption. In such cases, quenching may be relatively independent of fluorophore concentration when graphene surface availability is limiting, reinforcing the need for interference controls.

### 8.3 Resazurin (alamar blue) assay

Alamar Blue (AB) is a water-soluble, cell-permeable redox indicator used to track metabolic activity (often interpreted as viability), but it more directly reflects metabolism (Vieira-da-Silva and Castanho, 2023). Viable cells reduce non-fluorescent resazurin (blue) to fluorescent resorufin (pink) via mitochondrial and other cellular reductases (including diaphorases) using NAD(P)H-linked reducing capacity. The assay is typically read fluorometrically (Ex ∼530–570 nm; Em ∼580–620 nm). Absorbance readouts are possible (e.g., ∼570 nm with a reference ∼600 nm) but are less sensitive (O’Brien et al., 2000; Petiti et al., 2024). A decrease in fluorescence is generally interpreted as reduced metabolic activity/cytotoxicity; however, graphene materials can distort this relationship through assay interference (Riss et al., 2004).

Published AB outcomes for graphene materials remain contradictory (see Table 4). Some studies report decreased metabolic activity after exposure to G or GO (cell-type and material dependent), sometimes supporting the idea that surface functionalisation (e.g., GO vs. pristine G) reduces apparent toxicity. Others report little/no effect on macrophage viability across GO sizes. Other studies report apparent increases in fluorescence/metabolic activity, which may reflect biological stimulation, assay artefact, or both. Across these studies, differences in GO synthesis/source (Hummers-derived vs. commercial/other oxidation routes), and especially dispersion practices (sonication type, duration, and power) are repeatedly variable and inconsistently reported thereby limiting comparability.

**TABLE 4:** Fluorometric assays used to determine *in vitro* toxicity of different graphene family materials.

Assay	Material	Synthesis method	Comments	Cell line tested	Toxicity	References
Resazurin Alamar Blue assay (AB)	G, GO	Arc discharge	Functionalized by nitric acid	RAW 264.7	Yes – only with G	Sasidharan et al. (2012)
	GO	Hummer’s - derived from helical-ribbon carbon nanofibers (GANF 70% graphitization degree) by chemical oxidation	Dispersion - sonic probe for 20 min	PLHC-1, CLC	Yes – time dependent in CLC	Kalman et al. (2019)
	GO	Commercial – not specified	Dispersion - sonication at 50 W for 30 min	SH-SY5Y	Yes	Xiaoli et al. (2021)
	GO	Hummer’s	Different lateral sizes Dispersion - sonication for 45 min	HMDM	No	Rodrigues et al. (2018)
	GO	Commercial - not specified	Dispersion – bath sonication for 2 h at 37 kHz	Hep G2	Yes – dose dependent	Lammel et al. (2013)
	GO	Hummer’s	​	HMDM	Yes	Mukherjee et al. (2016)
	GO	Hummer’s	​	HEK293	Yes – dose dependent	Gurunathan et al. (2019)
Mitochondrial membrane potential – JC-1 assay	G, GO	Commercial - not specified	​	HaCaT	Yes	Pelin et al. (2018)
	G, rGO	Hummer’s	Reduction – chemical Dispersion - gentle sonication for 30 min in ultrapure water	U87-GM	Yes	Szczepaniak et al. (2018)
	GO	Hummer’s	Dispersion - sonication at 50 W for 30 min	HEK293	Yes – dose dependent	Gurunathan et al. (2019)
	GO	Commercial – not specified	​	SH-SY5Y	Yes	Xiaoli et al. (2021)
	GO, rGO	Hummer’s	Reduction - nicotinamide Dispersion – bath sonication for 1 min	MEF	Only for GO	Zhang and Gurunathan (2016)
H_2_DCFDA for ROS production	G	Catalytic chemical vapor deposition	​	PC12	Yes – dose and time dependent	Zhang et al. (2010b)
	GO	Hummer’s	Dispersion - bath sonication at 37 kHz for 2 h	A549	Yes – size dependent with large GO more toxic	Chang et al. (2011)
	GO	Hummer’s	​	HLF	Yes – dose dependent	Wang et al. (2013)
	GO	Hummer’s	​	BF-2	Yes – dose dependent	Srikanth et al. (2018)
	GO	Commercial – not specified	Dispersion - sonication at 50 W for 30 min	SH-SY5Y	Yes – dose dependent	Xiaoli et al. (2021)
	Carboxyl G, GO	Commercial - not specified	Dispersion – bath sonication for 2 h at 37 kHz	Hep G2	Yes – dose and time dependent	Lammel et al. (2013)
	G, GO	Hummer’s, bath or probe sonication at different time intervals for different sizes	Dispersion – bath and probe sonication	NHDF	Yes – dose dependent	Liao et al. (2011)
	Few layer G, GO	FLG – ball-milling, solvent free. GO-commercial and not specified	Reduction – chemical and heat Dispersion (sonication in ultra-pure water for 30 min)	HaCaT	Yes – dose dependent with GO more toxic	Pelin et al. (2018)
​	G, GO	Arc discharge	Functionalized by nitric acid	RAW 264.7	Yes – only with G	Sasidharan et al. (2012)
	G, GO	​	​	Caco-2/HT29	No	Domenech et al. (2020)
	GO, rGO	Commercial and sonication to produce different sizes	Reduction – hydrazine hydrate	HUVEC	Yes – GO more toxic than rGO and size dependent	Das et al. (2013)
	GO, rGO	Sonication - not specified	Reduction – hydrazine hydrate	A549, RAW246.7	Yes for GO but transient – decreased after 1 h	Horváth et al. (2013)
	GO, rGO	Hummer’s	​	Caco-2 cells	Yes – rGO more toxic than GO	Cebadero-Domínguez et al. (2022)
	GO, B-rGO	modified Hummer’s – bath and probe sonication	Reduction – biological (*B. marisflavi*)	MCF-7	Yes –B-rGO more toxic, both dose dependent	Gurunathan et al. (2013)
	GO, rGO	Hummer’s	Reduction - nicotinamide Dispersion – bath sonication for 1 min	MEF	Only for GO in dose dependent manner	Zhang and Gurunathan (2016)
	GO	Hummer’s	​	BEAS-2B	Yes – size dependent with large GO more toxic	Vranic et al. (2018)
	GO, GO- colloids	Commercial – not specified	No information	A549	Yes – GO more toxic	Domi et al. (2019)
	rGO	Hummer’s	Reduction – thermochemically and ascorbic acid Dispersion – 45 min at 44 kHz	16HBE14o	Yes with lower C/O ratios	Rodríguez-Garraus et al. (2023)

Because AB is fluorescence-based, graphene-driven quenching and adsorption are major concerns (Longhin et al., 2022). Kalman et al. (2019) recommended “interference controls” that include AB + nanomaterials without cells are useful but incomplete if they omit the key product controls, i.e., resorufin alone (no cells) and resorufin + nanomaterials (no cells). These are critical because graphene derivatives can quench resorufin fluorescence directly, which would bias results toward an underestimation of metabolic activity. Evidence exists that GO and carboxylated graphene can quench resorufin in a manner that may appear independent of fluorophore concentration when graphene surface availability is limiting (Lammel et al., 2013). By contrast, acellular incubations of AB reagent with graphene that show “no interference” only indicate that the material may not chemically reduce resazurin to resorufin under those conditions; they do not rule out quenching/adsorption of resorufin in cellular assays (Mukherjee et al., 2016).

### 8.4 Mitochondrial membrane potential (MMP)

Mitochondrial membrane potential (ΔΨm) is central to cellular energy metabolism and is commonly used as a sensitive indicator of early cellular stress and apoptosis (Sedlackova and Korolchuk, 2019). Fluorescent mitochondrial probes can discriminate high-ΔΨm from depolarised mitochondria and are widely used in nanotoxicology.

#### 8.4.1 JC-1 assay

JC-1 is a lipophilic cationic dye used to assess ΔΨm. In healthy/high-ΔΨm mitochondria, JC-1 forms aggregates (orange-red emission; ∼590 nm when excited ∼510 nm). Whereas, in depolarised/low-ΔΨm mitochondria (e.g., apoptotic cells), JC-1 remains monomeric (green emission; ∼527 nm). Readouts are typically microscopy, flow cytometry, or plate readers using appropriate filters, often expressed as a red/green ratio (Elefantova et al., 2018).

Multiple studies (see Table 4) report ΔΨm depolarisation in response to GO, FLG, G, and/or rGO in diverse cell models (keratinocytes, glioblastoma, kidney cells, neuroblastoma), with effects depending on material type and, in some cases, reduction chemistry (e.g., GO vs. NAM-rGO). As elsewhere, studies differ markedly in material provenance/synthesis, reduction approaches for rGO, and dispersion protocols (sonication often poorly quantified or absent).

Interference testing is sometimes attempted by incubating graphene materials with JC-1 in acellular systems, but results are not always clearly presented (Pelin et al., 2018). Given that carbon nanomaterials can interfere with fluorescence assays (including via intrinsic absorbance/fluorescence and quenching), it remains plausible that carbon-based materials (i.e., SWCNTs) may also distort JC-1 readouts (Holt et al., 2017). This risk is heightened because JC-1 relies on two emission channels and ratiometric interpretation.

### 8.5 H_2_DCFDA/DCFH-DA assay for reactive oxygen species

H_2_DCFDA (2′,7′-dichlorodihydrofluorescein diacetate) and carboxy-H_2_DCFDA are cell-permeable, non-fluorescent probes that are deacetylated intracellularly and then oxidised by reactive oxygen species (ROS) to fluorescent DCF (or carboxy-DCF) (Kim et al., 2005). The assay is widely used as an indicator of “overall oxidative activity,” but it is best interpreted cautiously as it reflects probe oxidation chemistry rather than a single ROS species (Wardman, 2008).

DCF-based ROS results are equally highly variable (Table 4). Many studies report dose- and time-dependent ROS increases after GO exposure across mammalian and fish cell models e.g., (Lammel et al., 2013; Wang et al., 2013), and sometimes after G/FLG exposure (Zhang Y. et al., 2010; Pelin et al., 2018). Size effects are reported in some systems e.g., larger GO inducing higher ROS under certain serum conditions; e.g., (Chang et al., 2011), but patterns are not universal. Comparisons between GO and rGO commonly report lower ROS with rGO than GO, although exceptions exist (including cases where rGO induces higher ROS than GO). Reduction chemistry matters: biologically or chemically reduced rGO variants sometimes induce ROS differently from GO, and some reduced forms (e.g., NAM-rGO in one study) show minimal ROS effects relative to GO (Zhang and Gurunathan, 2016). Methodological heterogeneity is extensive: GO is often Hummer’s-derived (or commercially sourced with unknown oxidation history), while rGO may be produced via hydrazine, biomass-mediated reduction, nicotinamide, ascorbate, thermal methods, etc., each altering defect density, oxygen content, and surface chemistry (Table 4).

Graphene materials can reduce the apparent DCF signal in acellular systems, consistent with quenching and/or adsorption of probe and/or product. This can lead to systematic underestimation of cellular ROS. Cell-free incubation of graphene materials with DCFDA can show signal suppression at higher graphene doses (Pelin et al., 2018). Physical adsorption of H2DCFDA and/or fluorescent DCF has been proposed as a major mechanism (Creighton et al., 2013). According to Bass et al. (1983) intracellular adsorption is particularly problematic because intracellular probe concentrations can be far higher than extracellular levels, potentially amplifying adsorption artefacts. At equal mass dose, materials with higher effective surface area and more graphitic domains (e.g., FLG vs. GO in some reports) may be stronger quenchers, again biasing toward under-reporting ROS. These findings support the need for interference controls that distinguish: (i) probe adsorption, (ii) product adsorption/quenching, and (iii) optical masking by the material itself.

## 9 Luminometric assays

Luminometric assays provide a stable glow-type signal after reagent addition and are widely used for proliferation/cytotoxicity screening (Niles et al., 2009).

### 9.1 ATP assay

Cellular ATP is a sensitive marker of viability because ATP synthesis collapses with severe cellular damage and membrane integrity loss (Riss et al., 2004). Luciferase-based assays quantify ATP via luciferin oxidation to oxyluciferin in an ATP- and Mg^2+^-dependent reaction, where luminescence intensity correlates with ATP content (Mueller et al., 2004) and, by extension, viable cell number (Lomakina et al., 2015).

ATP assays generally show concentration-dependent ATP depletion in some models exposed to GO or rGO (Xiaoli et al., 2021; Chortarea et al., 2022). In comparative rGO sets with different C/O ratios, higher oxygen content (lower C/O) is often associated with greater toxicity and ATP reduction, whereas highly reduced/high C/O materials may show weaker effects (Rodríguez-Garraus et al., 2023). Again, most studies use Hummers-derived GO (or commercial GO), and dispersion conditions commonly include sonication—though reporting remains inconsistent (Table 5).

**TABLE 5:** Luminometric assays used to determine *in vitro* toxicity of different graphene family materials.

Assay	Material	Synthesis method	Comments	Cell line tested	Toxicity	References
ATP assay	rGO	Arc discharge	Functionalized by nitric acid	HL-60	Yes – dose dependent	Chortarea et al. (2022)
	GO	Hummer’s	​	HEK293	Yes	Gurunathan et al. (2019)
	GO	Commercial – not specified	Dispersion - sonication at 50 W for 30 min	SH-SY5Y	Yes – dose dependent	Xiaoli et al. (2021)
	rGO	Hummer’s	Reduction – thermochemically and ascorbic acid Dispersion – 45 min at 44 kHz	16HBE14o	Yes with lower C/O ratios	Rodríguez-Garraus et al. (2023)

Nanomaterial interference in ATP assays is well recognised, often via adsorption of luciferin/luciferase components or light attenuation (Sibag et al., 2015). Carbon nanomaterials (e.g., CNTs) are documented interferents (Szymański et al., 2020). Although some graphene studies report no measurable interference (Chortarea et al., 2022), it is important to keep in mind that negative findings depend strongly on how interference was tested (e.g., inclusion of luciferin + material controls, signal recovery tests, and optical masking checks).

## 10 Synthesis, processing, and sonication as sources of contradictory toxicity results

Based on the available literature it is clear that materials labelled “G”, “GO”, and “rGO” are not equivalent across studies, because preparation routes strongly alter intrinsic properties (oxygen content/type, defects, edges, thickness/layers, contaminants), which then control surface behaviour and assay interactions. Some key aspects that stand out are:

*Graphene and GO preparation*: there are different methods used to prepare graphene from graphite. These include mechanochemical activation (León et al., 2011) and electrolytical exfoliation (Wang et al., 2009). For GO preparation, Hummers (Hummers and Offeman, 1958), modified Hummers (Rhazouani et al., 2021) and other oxidation routes yield different oxygen functionalities and defect profiles; toxicity can vary with oxidation chemistry (Chng and Pumera, 2013).

*rGO reduction*: hydrazine (Horváth et al., 2013), nicotinamide (Zhang and Gurunathan, 2016), biological (Gurunathan et al., 2013; Muthoosamy et al., 2015), ascorbate (Fernández-Merino et al., 2010; Zhang J. et al., 2010; Jaworski et al., 2015), sulfur-based reductants and thermal routes (Szczepaniak et al., 2018; Chortarea et al., 2022) generate rGOs with different oxygen removal, vacancy/edge defects, thickness/restacking, and residual impurities—changing both biological interactions and dye interference.

*Sonication is a major confounder* (during and after synthesis): it alters lateral size, thickness, defect density, oxidation state, edge chemistry, wrinkling, and potentially introduces contaminants or thermal effects (Taurozzi et al., 2011; Gonçalves et al., 2014; Mullick Chowdhury et al., 2014; Baig et al., 2018; Jiang et al., 2021). Over-sonication can damage sheets, change functional group distributions, and increase defect-rich reactive sites (Arao and Kubouchi, 2015; Mellado et al., 2019; Sieradzka et al., 2021). Because these same properties drive adsorption/quenching and optical masking, sonication can change both true bioactivity and assay artefact risk.

## 11 The role of functional groups

Exfoliated graphene oxide (GO) sheets carry abundant oxygen-containing functional groups, including epoxy, carbonyl, hydroxyl, and carboxyl moieties (Sun, 2019; Jiříčková et al., 2022). When evaluating the toxicity of graphene materials with dye-based assay systems, the presence, type, and density of these functional groups are critical because they can interact with assay reagents and products, contributing to contradictory findings in the literature. As outlined earlier, oxygenated functionalities can promote assay interference through mechanisms such as adsorption/absorption of dyes and fluorescence quenching.

### 11.1 Functional groups: dye adsorption in relation to size

Pristine graphene lacks surface functional groups, whereas GO and rGO carry oxygenated groups (e.g., –OH, –COOH, C=O) on basal planes and edges (Moon et al., 2015; Sun, 2019; Jiříčková et al., 2022). These functionalities create reactive/interactive sites that facilitate adsorption of inorganic and organic species. For example, dissociation of surface–COOH to–COO^-^ can enhance binding of cationic species, and carbonyl groups can contribute to chelation via oxygen lone pairs. GO’s heterogeneous 2D carbon–oxygen framework provides multiple interaction modes with dyes (Moon et al., 2015), including electrostatic attraction, hydrogen bonding, and π–π interactions (Lu et al., 2018). Consequently, GO is widely reported as a strong adsorbent for dyes, and reported adsorption capacities span very large ranges depending on dye chemistry and GO properties, e.g., acridine orange (Fiallos et al., 2015), methylene blue (Zhang et al., 2011), and methyl orange (Gong et al., 2017).

Several studies indicate that adsorption is strongly influenced by flake size and surface area. Smaller GO (higher surface area per mass) can provide more adsorption sites (Han et al., 2023), and differences in the proportion and distribution of functional groups (e.g., hydroxyl-rich samples) can translate into higher adsorption performance (Chen et al., 2015; Zhang S. et al., 2016). Conversely, other work suggests that larger GO architectures (e.g., aerogels assembled from large GO flakes) may show improved absorptivity/uptake behaviour relative to smaller-flake precursors (Guo et al., 2018), emphasizing that “size effects” depend on whether one is considering single flakes vs. assembled porous structures, and on how adsorption is quantified. Earlier warnings in the nanotoxicology literature noted that high-surface-area carbon materials can strongly adsorb small molecules and quench fluorescence of adsorbed probes, producing artefacts in dye-based assays (Wörle-Knirsch et al., 2006; Casey et al., 2007). Importantly, adsorption also modifies the effective surface of the graphene material during testing, potentially changing subsequent interactions (including with cells and assay components). Layer number further complicates adsorption: multilayer structures can increase uptake by providing interlayer trapping/entrapment (He et al., 2013; Travlou et al., 2013; Wang et al., 2015; Obraztsova et al., 2020), and reduction/chemical transformations of specific groups (e.g., carbonyl → hydroxyl in some *in situ* processes) have been proposed to increase the density of active sites (Sun et al., 2012).

### 11.2 Functional groups: surface charge, and hydrophilicity/hydrophobicity in relation to toxicity

Oxidative functionalisation changes graphene surface polarity and dispersibility (Chatterjee et al., 2015; Majeed et al., 2017). Pristine graphene is predominantly hydrophobic, whereas GO becomes more hydrophilic due to oxygenated groups (including negatively charged carboxylates at edges). These features influence dispersion stability in exposure media and affect how materials interact with proteins, membranes, and cells. Several studies emphasise that hydrophilicity/dispersibility in the exposure medium is a key determinant of bioavailability and membrane interaction, which can modulate cytotoxic potential (Cote et al., 2009; Hasan et al., 2010; Yang et al., 2010; Hsieh and Chen, 2011). Some reports propose that lower positive surface charge (or reduced effective charge interactions with cells) corresponds to milder apparent toxicity, highlighting the role of surface charge in cell–material contact and uptake (Wang et al., 2013).

### 11.3 Functional groups: contamination in relation to toxicity

Endotoxin contamination is a major confounder for carbon-based nanomaterial toxicology, particularly in immune-competent models (Vallhov et al., 2006). Contaminated materials can trigger inflammatory/toxic responses that are attributable to endotoxin rather than the nanomaterial itself, masking true effects or generating false positives (Li et al., 2017). To further substantiate the role of endotoxin in the toxicity outcomes, Lahiani et al. (2017) have assessed the toxicity of MWCNTs and graphene on macrophages using the LDH assay. Results have shown that both pyrogenated graphene and MWCNTs produced more LDH, indicating greater cell toxicity after 48-h incubation while depyrogenation reduced the toxicity of all tested materials. This was said to be due to the interaction of the surfaces of MWCNTs and graphene with endotoxin. Additional complexity arises because graphene-based materials may interfere with traditional endotoxin detection assays, motivating the development of alternative testing approaches (e.g., macrophage activation tests (Jasim et al., 2016; Mukherjee et al., 2016; Li et al., 2017). Despite this, relatively few studies explicitly report the use of endotoxin-free graphene materials, making it difficult to exclude endotoxin-driven artefacts when interpreting “toxicity” outcomes (Vranic et al., 2018; Chortarea et al., 2022).

Sample purity is another key driver of contradictory results. Oxidation and reduction routes can leave residual soluble reagents or introduce trace contaminants if washing and post-processing are insufficient (Pumera et al., 2012; Yin et al., 2015). GO synthesis in particular can involve oxidants and acids that may leave residues (Kim F. et al., 2010; Kalman et al., 2019), and metal impurities may originate from graphite stocks or reagents (Brisebois and Siaj, 2020). Trace metals and other ions associated with common reagents) can leach from materials and contribute to cytotoxicity (An et al., 2015), creating the appearance of graphene-driven toxicity when the response is partly or largely impurity-driven. Accordingly, comprehensive reporting of toxicology studies should include purity metrics and impurity screening, because high-purity GO produced via improved routes has been reported to show substantially reduced cytotoxic/inflammatory responses compared with more contaminated preparations studies (Sanchez et al., 2012; Li et al., 2017; Rodrigues et al., 2018). The limited attention to purity across much of the literature remains a serious limitation for cross-study comparisons.

## 12 Interference of G, GO, and rGO with different assay systems

A substantial body of literature now acknowledges that G, GO, rGO can interfere with many commonly used *in vitro* assay systems, particularly those relying on optical readouts (absorbance, fluorescence, luminescence). Many investigators have therefore included “interference controls”; however, these controls are frequently insufficient, because they test interaction with the substrate dye only and not with the assay product, or they assume that washing steps remove graphene sufficiently to prevent contact with probes. A key determinant of whether interference occurs is where the probe and its product reside (extracellular medium, plasma membrane-associated, intracellular cytosol, or within organelles) and whether graphene is present in those compartments. For example, washing cells before adding probes is widely used to minimize probe–nanomaterial interaction in the extracellular space. However, this does not necessarily prevent interactions with graphene that remains adsorbed to the cell membrane or that has been internalized, and therefore probe–graphene interactions may still occur after washing (Hirsch et al., 2011; Sasidharan et al., 2012).

### 12.1 Tetrazolium assays (MTT/MTS/WST family)

A recurring limitation is the use of cell-free incubation of graphene with tetrazolium substrates (e.g., MTT, MTS, WST-8) as an “interference test.” For example (Chortarea et al., 2022), dispersed rGO in cell-free medium and mixed it with assay reagents to exclude interference. While such experiments can detect direct catalytic reduction of substrate (if it occurs), they cannot evaluate adsorption/interaction with the assay product when product formation requires viable cells. Horváth et al. (2013) incubated MTT solution with GO/rGO and found no evidence of catalytic conversion. However, in the absence of cells, formazan is not generated, so adsorption/quenching/optical masking effects on formazan cannot be assessed in that configuration. A more appropriate interference test would include pre-formed formazan (or an equivalent product standard) and graphene materials under assay-relevant conditions. Srikanth et al. (2018) replaced exposure medium with fresh MTT-containing medium after GO exposure and then transferred supernatant for absorbance reading. This does not necessarily prevent interference because graphene may remain membrane-bound or intracellular, and may still adsorb product or contribute to optical masking. Similarly, many studies rely on “untreated cells” as negative controls and perform cell-free tests as positive controls (Zhang Y. et al., 2010; Wang et al., 2013; Nguyen et al., 2015; Srikanth et al., 2018; Domi et al., 2019), which does not resolve product-level interactions. Cell-free incubation with WST-8 substrate has been used (Liao et al., 2011; Gurunathan et al., 2013; Nguyen et al., 2015), but again does not test interactions with the formazan product, which is the species quantified. Many “interference controls” in tetrazolium assays can rule out (some) substrate reduction in acellular settings, but they generally cannot exclude adsorption of product, optical absorption/scattering artifacts, or fluorescence/absorbance quenching that occur under true assay conditions.

### 12.2 ATP (luciferase/luciferin) assays

ATP assays depend on luciferase-mediated oxidation of luciferin in the presence of ATP to produce a luminescent signal. Testing graphene + CellTiter-Glo reagent in cell-free medium (Chortarea et al., 2022) may not capture the relevant interference pathways if the primary interaction is with the light-emitting reaction output (e.g., signal attenuation) or with components under ATP-present conditions. A stronger interference assessment would include controls with known ATP concentrations (or ATP standards) and graphene present, to test whether signal generation is altered under ATP-relevant conditions.

### 12.3 Resazurin (alamar blue) assays

Mukherjee et al. (2016) incubated graphene materials with AB reagent in an acellular system and observed no interference. However, conversion of resazurin to resorufin in this assay is mediated mainly by cellular metabolic activity (e.g., diaphorases using NAD(P)H), so acellular incubation does not generate the fluorescent product. Therefore, such tests cannot evaluate graphene interactions with resorufin, the quantified fluorophore. Product-focused controls (resorufin ± graphene, cell-free) are more informative for detecting fluorescence quenching or adsorption-based signal loss (Lammel et al., 2013; Kalman et al., 2019).

### 12.4 Neutral red uptake (NRU) assay

NRU quantifies the ability of viable cells to accumulate neutral red in lysosomes, followed by dye extraction and absorbance/fluorescence measurement. Controls that include only untreated cells and graphene-treated cells (e.g. (Muthoosamy et al., 2015)) do not directly test whether graphene interacts with neutral red (or its protonated/lysosomal form) in a way that alters extraction efficiency or optical readout. Product/probe interaction tests using relevant dye forms ± graphene (cell-free) are needed to probe quenching/adsorption mechanisms, as demonstrated in quenching-focused designs (Lammel et al., 2013).

### 12.5 Fluorescence quenching controls: partial solutions, not exclusions

Several investigators recognized fluorescence quenching as a major interference mechanism and proposed additional steps. Kalman et al. (2019) recommended (i) reading fluorescence of graphene-exposed cells before/after washing, and (ii) incubating cells with assay conversion products such as resorufin (AB) or 5-carboxyfluorescein (from CFDA-AM). While these approaches probe some aspects of signal distortion, they may still confound cell-dependent uptake/retention with material-dependent quenching. A clearer product-focused interference test is to incubate the assay products with graphene materials in the absence of cells, then quantify fluorescence loss attributable to adsorption/quenching. Lammel et al. (2013) implemented this approach for 5-carboxyfluorescein, resorufin, and protonated neutral red and observed dose-dependent attenuation of fluorescence, consistent with quenching/adsorption mechanisms (Lammel et al., 2013; Valdehita et al., 2023). Such experiments can confirm quenching potential, but they do not automatically “exclude” interference under all biological assay conditions; rather, they demonstrate that the assay is vulnerable without robust mitigation.

### 12.6 Dye-exclusion assays and flow-cytometry-based probes

#### 12.6.1 Trypan blue

Most Trypan blue studies use untreated cells as controls (e.g., (Chang et al., 2011; Jaworski et al., 2013; Hinzmann et al., 2014; Zhang and Gurunathan, 2016). This does not address the possibility that graphene retained on membranes or co-pelleted with cells may visually mimic staining or alter counting accuracy, potentially leading to misclassification (Altman et al., 1993).

#### 12.6.2 Annexin V/propidium iodide (PI)

Untreated-cell controls are similarly insufficient to exclude quenching effects. PI fluorescence can be quenched by GO via adsorption (Liu F. et al., 2014), and graphene materials are known quenchers of various fluorophores (Treossi et al., 2009; Kim J. et al., 2010). Therefore, product/probe-specific interaction controls remain essential.

### 12.7 ROS assays: DCFH-DA/DCF readout

Pelin et al. (2018) and Vranic et al. (2018) included cell-free controls by incubating graphene materials with the DCFDA probe over time, but did not assess interactions with the fluorescent product, DCF, under cell-free conditions. Since adsorption/quenching of DCF is a plausible interference mechanism, omission of DCF + graphene product controls limits interpretability. Other studies reporting concentration-dependent ROS increases [e.g. (Domi et al., 2019; Xiaoli et al., 2021)] similarly did not include DCF product controls, despite known masking/quenching phenomena reported in related work.

Despite the well-established impact of interference on experimental accuracy, a significant gap remains in the standardized reporting of rigorous controls and comprehensive interference assessments (Andraos et al., 2020). Current literature frequently cites a single control parameter and therefore fails to account for the multifaceted nature of different interference types. When multiple interference pathways occur simultaneously, they do not behave linearly. Calculating a corrected value for various overlapping mechanisms is unfeasible. In addition, interference is traditionally assessed in cell-free systems, however, the intracellular environment is more complex. The presence of cellular biomass can alter the degree of interference, making it impossible to assume that cell-free baselines are representative of *in vitro* conditions thereby further invalidating simple background subtraction or correction (Bancos et al., 2012; Ong et al., 2014; Labouta et al., 2018; Andraos et al., 2020). The exhaustive implementation of a full battery of interference controls is labour-intensive and demanding, leading to the widespread omission of proper controls in peer-reviewed research. Modern label-free advances in methodologies (discussed below) bypass any interference uncertainties and ensures data integrity without the need for complex compensatory calculations. Consequently, rather than expending effort on tedious and often omitted retroactive corrections, scientific focus should shift toward the adoption of inherently interference-free, label-free technologies and provide a more reliable, streamlined approach to predictive toxicology.

## 13 Interference-free tests for toxicity assessment of graphene family materials

Because many conventional cytotoxicity assays rely on optical detection of dyes or dye-derived products, graphene-mediated adsorption, quenching, or optical masking can directly distort assay readouts. This supports the adoption of interference-resistant or interference-free alternatives. Moreover, since the same types of graphene can still vary significantly in surface functionalization and oxidative state, among other physicochemical properties, their toxicological profiles and propensities to interfere with toxicity assays are equally inconsistent. Consequently, interference assessments must be conducted independently for every unique GFM under investigation. Low interference observed in one graphene oxide material cannot be used as a proxy for another graphene oxide material. The toxicity profiles and propensity for assay interference are further complicated by the fact that GFMs are rarely utilized as isolated entities. They are frequently integrated into complex matrices or nanocomposites (e.g., with polymers or metal nanoparticles). These surrounding matrices may further introduce assay variabilities. This inherent unpredictability further underscores the necessity of adopting label-free, interference-free methodologies, which offer a more robust analytical approach by remaining largely indifferent to these fine-scale physicochemical variations. Importantly, even with interference-free methods, the quality and comparability of dispersions must be controlled. Hydrophilic GO disperses more readily than pristine graphene, which is hydrophobic and prone to agglomeration in aqueous media (Bussy et al., 2013; 2015). Agglomeration alters delivered dose, cellular contact, uptake, and apparent toxicity (Pinto et al., 2013), meaning that “generalized toxicity profiles” without dispersion characterization can be misleading.

### 13.1 Colony formation (clonogenic) assay

The clonogenic (colony formation) assay measures the ability of a single cell to proliferate into a colony, avoiding reliance on dye-based optical endpoints. Interlaboratory comparisons have supported its low susceptibility to nanoparticle interference (Ponti et al., 2014). More recently, colony-forming efficacy (CFE) approaches have been proposed as more accurate than assays affected by nanomaterial-associated optical artifacts (Won et al., 2022), and they have been applied to GO to assess proliferation responses in human keratinocytes (Frontiñan-Rubio et al., 2022). Earlier work also highlighted its utility for carbon-based materials (Herzog et al., 2007).

### 13.2 Label-free impedance technology

Electrochemical impedance-based systems provide non-optical, real-time monitoring of cellular responses (ISO/TS 21633, 2021). Commercial platforms (e.g., xCELLigence®, CellSine, ECIS) quantify impedance changes in cell monolayers to infer viability, barrier integrity, and growth kinetics. The approach has been demonstrated and validated for nanoparticle toxicity screening (Otero-González et al., 2012; Vetten et al., 2013; Veschi et al., 2024) and has been applied to graphene-related materials (e.g. (Hondroulis et al., 2012; Male et al., 2012; Bonanni and Pumera, 2013; Bavorova et al., 2022)).

### 13.3 Metabolic flux analysis (OCR/ECAR)

Real-time oxygen consumption rate (OCR) and extracellular acidification/proton efflux measurements (e.g., Seahorse XF analyzers) provide label-free assessment of mitochondrial and glycolytic function. This approach has been used to evaluate metabolic effects of graphene materials in macrophages and other cell types (Georgieva et al., 2020; Povo-Retana et al., 2021; Alhourani et al., 2022).

### 13.4 Electron spin resonance (ESR/EPR) for ROS and radical detection

ESR/EPR directly detects short-lived radicals using spin traps, bypassing fluorescent dye readouts. It has been used to quantify carbon-centered radicals and hydroxyl radicals associated with GO chemistry, impurities, and H_2_O_2_ decomposition (Ge et al., 2012; Zhang W. et al., 2015; Vranic et al., 2018). Radical signatures are sensitive to oxidation level (C/O ratio), defect density, and edge chemistry, and can help disentangle oxidative mechanisms from assay artifacts.

### 13.5 Confocal live-cell imaging exploiting intrinsic graphene fluorescence

Some GO materials exhibit intrinsic fluorescence that can be exploited to monitor cell–material interactions without attaching external fluorophores (Vranic et al., 2018). This can support kinetic, single-cell observations, although it does not by itself replace cytotoxicity endpoints and must be interpreted alongside exposure characterization.

## 14 Conclusions and recommendations

Contradictory toxicity findings for graphene materials arise from multiple interacting variables: preparation route (including sonication during and after synthesis), lateral size, layer number, surface area, defect density/topography, impurities/endotoxin, radical-generating potential, surface charge, and—most importantly—surface chemistry (C/O ratio; type/degree of functionalization) (Sanchez et al., 2012; Bianco, 2013). Therefore, many apparent discrepancies across studies likely reflect differences in material properties and experimental handling rather than genuine disagreement in biological effects. Thus, detailed material characterization, transparent methodological reporting, and careful consideration of exposure conditions are essential prerequisites for meaningful interpretation and comparison of graphene toxicity data. These same factors govern the likelihood and magnitude of assay interference, including dye adsorption and fluorescence quenching, which has long been recognized as a source of misleading cytotoxicity outcomes for nanomaterials (Ribeiro et al., 2017).

Graphene materials (and related carbon nanomaterials such as CNTs) can adsorb probe dyes and/or assay products, alter optical signals, and remain associated with cells despite washing, leading to false positive or false negative toxicity estimates (Wörle-Knirsch et al., 2006; Casey et al., 2007; Davoren et al., 2007). Similar vulnerabilities have been reported for tetrazolium assays, fluorometric viability probes, LDH, and ATP bioluminescence systems, emphasizing that uncritical reliance on these methods may propagate unreliable mechanistic interpretations and inconsistent datasets.

Accordingly, this review supports two core recommendations. Firstly, when using colorimetric, fluorometric, and luminometric dye-based assays for G/GO/rGO it is essential to that rigorous, assay-specific interference testing is undertaken that includes product-focused controls (product ± graphene, cell-free), alongside dispersion characterization and appropriate washing/separation steps. Secondly, based on interference of graphene materials in all the assay systems shown in the previous sections, preference should be given to interference-free or interference-resistant endpoints, such as clonogenic assays, label-free impedance monitoring, metabolic flux analysis (OCR/ECAR), and ESR/EPR-based radical detection, to generate higher-confidence toxicology data suitable for mechanistic interpretation, predictive modelling, and integration into robust datasets (Robinson et al., 2016; Andraos et al., 2020).
